# Two-stage revision surgery with preformed spacers and cementless implants for septic hip arthritis: a prospective, non-randomized cohort study

**DOI:** 10.1186/1471-2334-11-129

**Published:** 2011-05-16

**Authors:** Carlo L Romanò, Delia Romanò, Enzo Meani, Nicola Logoluso, Lorenzo Drago

**Affiliations:** 1 Dipartimento di Chirurgia Ricostruttiva e delle Infezioni Osteo-articolari Istituto Ortopedico I.R.C.C.S. Galeazzi - Via Riccardo Galeazzi, 4 - 20161 - Milano - Italy; 2 Centro di Chirurgia Osteo-articolare Settica Istituto Ortopedico Gaetano Pini - Via G. Pini, 9 - 20122 - Milano - Italy; 3 Laboratorio analisi e microbiologia Istituto Ortopedico I.R.C.C.S. Galeazzi - Via Riccardo Galeazzi, 4 - 20161 - Milano - Italy

## Abstract

**Background:**

Outcome data on two-stage revision surgery for deep infection after septic hip arthritis are limited and inconsistent. This study presents the medium-term results of a new, standardized two-stage arthroplasty with preformed hip spacers and cementless implants in a consecutive series of adult patients with septic arthritis of the hip treated according to a same protocol.

**Methods:**

Nineteen patients (20 hips) were enrolled in this prospective, non-randomized cohort study between 2000 and 2008. The first stage comprised femoral head resection, debridement, and insertion of a preformed, commercially available, antibiotic-loaded cement hip spacer. After eradication of infection, a cementless total hip arthroplasty was implanted in the second stage. Patients were assessed for infection recurrence, pain (visual analog scale [VAS]) and hip joint function (Harris Hip score).

**Results:**

The mean time between first diagnosis of infection and revision surgery was 5.8 ± 9.0 months; the average duration of follow up was 56.6 (range, 24 - 104) months; all 20 hips were successfully converted to prosthesis an average 22 ± 5.1 weeks after spacer implantation. Reinfection after total hip joint replacement occurred in 1 patient. The mean VAS pain score improved from 48 (range, 35 - 84) pre-operatively to 18 (range, 0 - 38) prior to spacer removal and to 8 (range, 0 - 15) at the last follow-up assessment after prosthesis implantation. The average Harris Hip score improved from 27.5 before surgery to 61.8 between the two stages to 92.3 at the final follow-up assessment.

**Conclusions:**

Satisfactory outcomes can be obtained with two-stage revision hip arthroplasty using preformed spacers and cementless implants for prosthetic hip joint infections of various etiologies.

## Background

Primary septic arthritis in adults is a rare, but potentially devastating disease. The key factor in the selection of the type of surgery is symptom duration. Early onset of infection can be treated with radical open or arthroscopic debridement. Failure rates after debridement increase rapidly in the first days after onset of symptoms [1] and more radical surgery may be required as joint damage takes place. Resection hip arthroplasty helps to eradicate the infection but leaves the patient with a leg length discrepancy, dependency on ambulatory aids, and variable pain relief [2,3].

Two-stage revision with the use of an antibiotic-loaded acrylic cement spacer is a well-established procedure in the management of chronically infected total hip replacement [4-6]. Two-stage total hip arthroplasty (THA) with an interval antibiotic-loaded polymethylmetacrylate spacer has been recently proposed to clear infection and improve hip function after septic hip arthritis [7-9]. In a recent case report, Regis et al. [10] described successful two-stage hip reconstruction after septic arthritis with a commercially produced, preformed antibiotic-impregnated cement spacer.

The aim of this prospective, non-randomized cohort study was to assess the medium-term results in a consecutive series of patients with septic hip arthritis treated according to the same protocol that involved preformed, commercially available antibiotic-loaded cement spacers and cementless implants for two-stage reconstruction of the hip.

## Methods

The cohort consisted of 19 consecutive patients (total of 20 hips as both hips in 1 patient [FF in Table 1] were treated 2 years apart) referred to our departments from 2000 to 2008 for chronic deep hip joint infection. All patients had received one or more unsuccessful courses of systemic antibiotic therapy prior to coming to our observation. The indications for two-stage revision were: failure of conservative treatments; clinical and laboratory signs of persistent inflammation; functional impairment of the affected joint; and radiographic signs of joint damage (joint-line narrowing, subchondral osteolysis, bone loss and/or femoral head necrosis).

**Table 1:** Pre-operative patient characteristics

Patient	Sex	Etiology of infection	Age (yrs)	Habits and co-morbidities	Isolated microorganism
MZ	M	Post-osteosynthesis	42	Smoking - Drug abuse	MSSA
GS	M	Post-osteosynthesis	56	Smoking - Alcohol abuse	MRSA
MC	F	Post-osteosynthesis	30	/	MRSA
FS	F	Hematogenous	61	Diabetes	MSSA
MB	F	Intra-articular injection	63	/	MSSA
AP	M	Post-osteosynthesis	46	Smoking - Diabetes	Enterococcus
MO	M	Post-osteosynthesis	66	Smoking	MRSA
FF	F	Hematogenous (bilateral)	52	Radiotherapy - Lymphedema	MSSA
GB	F	Post-osteosynthesis	75	Diabetes	Neg
PI	F	Hematogenous	61	Smoking - Diabets	MSSA
GF	M	Hematogenous	32	Smoking - Diabetes	CoNS
CC	M	Hematogenous	64	Vasculopathy	Neg
MR	M	Post-osteosynthesis	47	/	Neg
TS	M	Post-osteosynthesis	61	/	MRSA
LL	F	Post-osteosynthesis	39	Diabetes	*Pseudomonas aeruginosa*
GR	F	Hematogenous	66	Rheumatoid arthritis	MSSA
DA	M	Post-osteosynthesis	66	/	CoNS
GL	F	Hematogenous	55	Lymphoma	Neg
PQ	F	Post-osteosynthesis	77	Diabetes	CoNS

Abbreviations: MSSA: methicillin-sensitive *Staphyloccus aureus*; MRSA: methicillin-resistant *Staphyloccus aureus*; CoNS: coagulase-negative staphylococci; Neg: negative culture.

All patients provided written consent to participate in the study and permission to publish clinical images. The ethics committee of Local Health Authority 1, Milan, Italy approved the study protocol.

Infection was diagnosed according to the criteria set forth by Spangehl et al.: at least three positive results for erythrocyte sedimentation, C-reactive protein, aspiration, frozen section or intraoperative cultures [11].

Clinical, laboratory and radiographic assessments were performed by an independent observer pre-operatively at the time of spacer removal, and at the last follow-up after reimplantation. Epidemiological data were gathered using a standard data collection form.

Clinical assessment was performed using a visual analog scale (VAS) (a 100-mm scale where 0 indicates no pain and 100 maximum tolerable pain) and the Harris Hip score [12]. Clinical signs of recurrent infection (redness, swelling, pain, fistulae) were recorded at follow-up.

Laboratory tests included complete blood count with differential, erythrocyte sedimentation rate, C-reactive protein, urea and creatinine, and creatinine clearance test.

Plain radiographs were obtained in anteroposterior and translateral views of the hip joint. Radiographic examination was performed pre-operatively, at spacer removal, at reimplantation, at 3, 6 and 12 months postoperatively, and then yearly thereafter.

The primary outcomes were the rates of infection recurrence, re-revision for infection and aseptic prosthesis loosening, and clinical outcome at the last follow-up after revision. The secondary outcomes were eradication rate of infection, spacer integrity and stability, and clinical outcome at the time of spacer removal.

The surgical procedure was similar in all patients in this series. All were operated by one of us (CLR or EM) through a lateral approach, with the patient in the supine position in both the first- and the second-stage operations.

**Figure 1:**
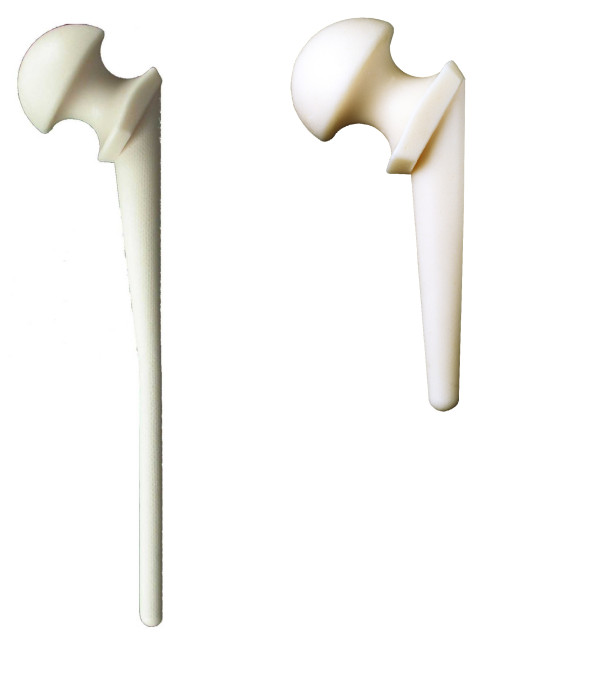
**The preformed spacer used in the study**. The preformed spacer comes in two stem lengths and three head sizes (not shown).

After radical synovectomy, debridement and excision of all infected parts, the femoral head was resected onto healthy bone and the acetabular cavity gently reamed. Synovial fluid and synovium were obtained for culture. After debridement, the joint was rinsed with approximately 10 liters of saline solution. After reaming of the femoral canal, a preformed antibiotic-loaded hip spacer (InterSpace^® ^Hip, Tecres SpA, Verona, Italy - Hexactech Inc. Gainesville, FL, USA) (Figure 1) was implanted. The InterSpace^® ^Hip is an off-the-shelf polymethylmetacrylate antibiotic-loaded pre-formed hip spacer. The inner part of the spacer features a stainless steel rod to enhance mechanical resistance. The cement is pre-loaded by the manufacturer with gentamicin (1.9%). The InterSpace^® ^Hip comes in three different head sizes and two stem sizes, short (260 mm) and long (360 mm), which may be intraoperatively chosen on the basis of femoral bone loss and the need for distal fixation of the implant.

To prevent implant rotation, the spacer was fixed only in the proximal part with one pack of antibiotic-loaded cement (Cemex Genta, Tecres) containing gentamicin (1.9%) and vancomycin (5%). The vancomycin powder was thoroughly mixed with the cement powder to obtain a fine consistency before adding the liquid monomer. No bone grafts were used at the time of spacer implantation. All patients received a minimum of 4 weeks of organism-specific antibiotics postoperatively and returned for clinical follow-up at the completion of their antibiotic course.

Patients with successful eradication of infection, as evidenced clinically and by complete blood count with differential and C-reactive protein within the normal range, underwent the second stage of hip joint reconstruction. In cases of clinical suspicion of persistent infection, the hip joint was aspirated before reimplantation and samples were obtained for culture and white blood cell count. Intraoperative cultures were obtained at the second stage procedure in all cases. At revision, the hip was exposed through the same lateral incision and the spacers were removed.

Revision was performed with modular titanium cementless femoral components (Profemur, Wright, AEQUA, AdlerOrtho or S-ROM, Johnson&Johnson DePuy). Unconstrained cementless acetabular components were used in all cases. Partial weight bearing with two-crutches was allowed for 6 weeks, then one-crutch walking for 4 to 6 more weeks. Full weight bearing was permitted at 10 to 12 weeks after surgery. Systemic antibiotics were administered for 4 weeks after the operation. No drains were placed after either procedure.

All patients received enoxaparin 0.4 ml/die for 30 days after surgery to prevent thromboembolic complications and celecoxib 200 mg/die for 14 days after revision surgery to prevent heterotopic ossifications [13].

## Results

Table 1 reports patient demographics and pre-operative characteristics. Of the total of 19 patients, 14 (73%) were identified as type-B hosts, i.e., patients with one or more risk factors for infection (Table 1), according to the Cierny-Mader classification [14]. There was a mean 5.8 ± 9.0 months between the diagnosis of infection and the index-surgery. Four out of 19 (21%) patients received one surgery (two open and two arthroscopic, respectively) after diagnosis of infection and prior to spacer implantation.

In 11 patients, joint aspiration was performed prior to surgery at our institution; none of these patients presented with draining fistulas and previous cultures were either not available or negative.

At least 5 different tissue samples were taken perioperatively in all the cases. Cultures were positive in 16 of 20 hips (80%). *Staphylococcus aureus *and coagulase-negative staphylococci (CoNS) (13/20) were predominantly identified (Table 1).

The duration of postoperative antibiotic treatment after spacer implantation was from 4 to 6 weeks (mean 5.2 ± 1.1); methicillin-sensitive gram-positive bacteria were treated with a combination of amoxicillin/clavulanic acid or cephalosporins and levofloxacin, or trimethoprim/sulfamethoxazole or rifampicin; methicillin-resistant bacteria were treated with a combination of a glycopeptide (vancomycin or teicoplanin) with either levofloxacin or trimethoprim/sulfamethoxazole or rifampicin. Patients with negative culture results were treated empirically with a combination of vancomycin or teicoplanin and levofloxacin; the patient with *Pseudomonas aeruginosa *infection was treated with meropenem and levofloxacin according to the antibiogram.

Joint aspiration was also performed in two patients with clinical suspicion of persistent infection prior to spacer removal; the culture tested negative in both cases and the leukocyte count was 600/uL and 480/uL, respectively.

All 20 hips were successfully converted to THA an average 22.3 ± 5.1 weeks after spacer implantation. Complications included two spacer dislocations, one transient femoral nerve palsy, and two deep vein thrombosis. At the time of spacer removal, a single intraoperative specimen gave a positive culture result in two patients (coagulase-negative bacteria in both cases), without pre-operative signs of infection. No alteration in the routine postoperative protocol as regards antibiotic treatment duration (4 weeks) was made in either case; the choice of antibiotics (vancomycin and levofloxacin in both cases) was decided according to the results of antibiogram testing of the bacteria isolated from intraoperative cultures.

At a mean follow-up of 56.6 months (range, 24 - 104), none of the patients was lost, but one died four years after surgery in a road car accident. One patient (AP) developed recurrence of infection associated with sinking of the femoral stem which required re-revision two years after intervention. This patient was a heavy smoker with a history of diabetes, post-traumatic hip infection, associated with osteomyelitis of the proximal third of the femur, and had presented with draining fistulas and enterococcus infection.

None of the remaining patients required reintervention; they showed no local or general signs or laboratory data of infection and never had to restart antibiotics at any point.

The average VAS pain score was 48 ± 20 (range, 35 - 84) pre-operatively, 18 ± 15 (range, 0 - 38) prior to spacer removal, and 8 ± 10 (range, 0 - 15) at the last follow-up after prosthesis implantation. The mean Harris Hip score was 27.5 ± 15.3 pre-operatively, 61.8 ± 18.6 at the time of spacer removal, and 92.3 ± 17.4 at the final follow-up assessment.

A leg length discrepancy between 1 and 2 cm was observed in three patients and less than 1 cm in the remaining patients.

**Figure 2:**
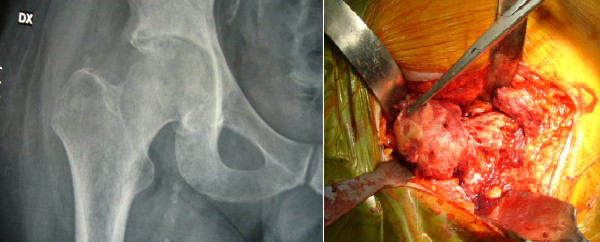
**Clinical case (patient GF)**. Pre-operative X-ray and intraoperative finding. Hematogenous septic hip arthritis.

**Figure 3:**
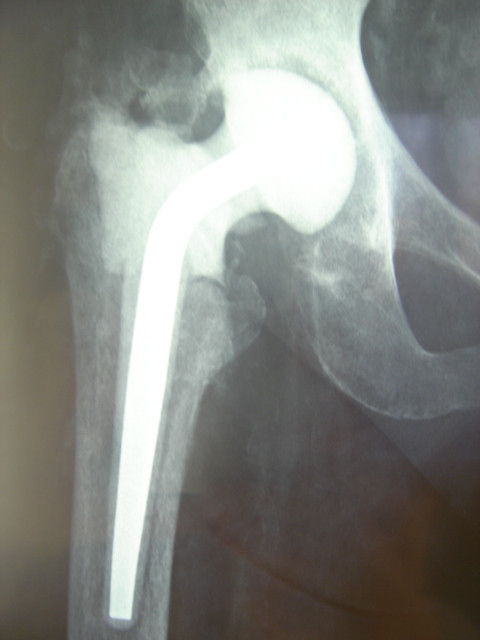
**Clinical case (patient GF)**. Postoperative X-ray prior to hip spacer removal.

**Figure 4:**
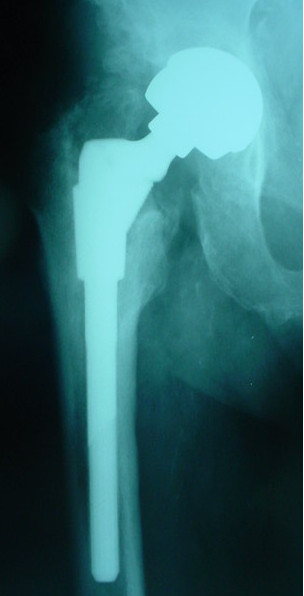
**Clinical case (patient GF)**. Radiographic control four years after cementless hip prosthesis implantation.

No hip prosthesis dislocation occurred. No component showed migration or osteolysis at radiographic evaluation (Figures 2, 3 and 4).

## Discussion

Based on the most recent literature, staged reconstruction of the hip after septic arthritis may be considered as a reliable alternative to conventional resection arthroplasty. However, owing to the limited series and the differences in technical approach, the results with this procedure appear unpredictable and difficult to standardize.

Chen et al. [15] reported a re-infection rate of 14% and a complication rate of 36% after two-stage THA without temporary devices for primary septic arthritis of the hip. Morshed et al. [16] described a case of post-injection septic arthritis of the hip successfully managed with the implantation of an antibiotic-impregnated cement block and a hip prosthesis after failed previous surgical debridement and partial femoral head resection. A more recent case report was published by Barrett and Bal [17]; very recently, Regis et al. [10] were the first to report on another single case of hematogenous infection successfully treated with a preformed hip spacer and a cementless hip prosthesis.

Apart from these case reports, as far as we know, only five papers have described a series of patients treated with a temporary device and a hip prosthesis in the second stage for septic hip arthritis. Schoellner et al. [18] were probably the first to report a preliminary experience with five individually manufactured bone cement spacers for septic hip revision. They highlighted the adaptability of this mechanically tested stem and discussed its indication in primary joint infection without giving additional details. The relevant data from the four other more detailed papers are reported in Table 2 and compared with those of the present study. The infection recurrence rate after prosthesis implantation ranges from 0 to 15%. This latter result was reported by Bauer et al. [19], who recently described a retrospective mixed series of patients treated with either one- or two-stage procedures after hip or knee septic arthritis. In this paper, "evolutive septic arthritis" (17 knees and 13 hips) was treated with a two-stage procedure. The short time interval between stages (mean, 6 weeks) and the absence of antibiotic in the cement spacer may explain the relatively high incidence of infection recurrence.

**Table 2:** Data from the literature.

Study	Number of hips (patients)	Follow-up (average, months)	Time to spacer removal (weeks)	Reinfection rate (at spacer removal)	Reinfection rate (after total hip replacement)
Diwanji et al., 2008	9 (9)	42	23	1/9 (12%)	1/9 (12%)

Kelm et al., 2009	8 (8)	12	12	0/8 (0%)	0/8 (0%)

Huang et al., 2010	15 (14)	42	13	1/15 (6.7%)	0/15 (0%)

Bauer et al., 2010	13 (13)	60	6	0/13 (0%)	2/13 (15%)

This study	20 (19)	56	22	0/20 (0%)	1/20 (5%)

**Pooled data**	**65 (63)**	**42 ± 19**	**15 ± 7 **	**2/65 (3.1%)**	**4/65 (6.1%)**

Two-stage hip prosthesis for septic arthritis of the hip. Comparison of results between studies and pooled data.

Our results are in line with previously published data at a longer follow-up and in a larger patient series. Ours is also the first paper to document, in a prospectively followed patient cohort, the safety and efficacy of a preformed spacer and cementless hip prosthesis.

The most relevant clinical advantage of using an antibiotic-loaded spacer is that it helps to maintain joint space and minimizes the risk of large limb shortening, while local antibiotic delivery prevents bacterial re-colonization of the implant [4-10]. Furthermore, preformed antibiotic-loaded spacers offer off-the-shelf availability, a standardized and reproducible technique, known mechanical resistance [20,21], predictable antibiotic release [22] and shorter operating time [23,24], being available in short and long stemmed shapes [25] that can be chosen intraoperatively based on femoral bone loss.

The choice of antibiotics loaded to the cement spacer and administered systemically in this series was clearly effective, with proper surgical technique, in eradicating infection in patients with septic hip arthritis due to different bacteria, including methicillin-resistant strains. However, with the growing occurrence of multi-drug and vancomycin-resistant strains, the use of more recently available antibacterial agents may be necessary in selected cases. The efficacy of daptomycin or linezolid in treating septic arthritis is currently under study [26,27].

The main limitations of the present study are the limited sample size, the lack of a comparator group, and the relatively short duration of follow-up. Due to the relatively low prevalence of septic arthritis of the hip, coordinated multicenter randomized trials are needed to evaluate long-term outcomes after two-stage revision with this technique.

## Conclusions

Two-stage total hip arthroplasty with preformed hip spacers and cementless implants provide a reliable solution in the medium-term follow-up for septic arthritis of the hip and may be offered to patients as a valuable treatment option.

## Competing interests

The authors declare that they have no competing interests.

## Authors' contributions

CLR and EM designed the study, performed the surgeries and drafted the manuscript. DR and NL collected and processed the data. LD revised the manuscript and performed laboratory testing and microbiological analysis. All authors revised the manuscript critically, and all approved the final version that was submitted.

## Pre-publication history

The pre-publication history for this paper can be accessed here:

http://www.biomedcentral.com/1471-2334/11/129/prepub
